# Direct measurement of Bisphenol A (BPA), BPA glucuronide and BPA sulfate in a diverse and low-income population of pregnant women reveals high exposure, with potential implications for previous exposure estimates: a cross-sectional study

**DOI:** 10.1186/s12940-016-0131-2

**Published:** 2016-04-12

**Authors:** Roy R. Gerona, Janet Pan, Ami R. Zota, Jackie M. Schwartz, Matthew Friesen, Julia A. Taylor, Patricia A. Hunt, Tracey J. Woodruff

**Affiliations:** Program on Reproductive Health and the Environment, Department of Obstetrics, Gynecology and Reproductive Sciences, University of California, Mailstop 0132, 550 16th Street, 7th Floor, San Francisco, CA 94143 USA; Department of Environmental and Occupational Health, Milken Institute School of Public Health, George Washington University, 950 New Hampshire Avenue NW, Suite 414, Washington, DC 20052 USA; School of Molecular Biosciences and Center for Reproductive Biology, Washington State University, PO Box 647521, Pullman, WA 99164-7521 USA; Division of Biological Sciences, University of Missouri-Columbia, 114 Lefevre Hall, Columbia, MO 65211 USA

**Keywords:** Bisphenol A, Pregnant women, Children’s environmental health, Exposure sources, Liquid chromatography-tandem mass spectrometry (LC-MS/MS)

## Abstract

**Background:**

Bisphenol A (BPA) is a ubiquitous, endocrine-disrupting environmental contaminant that increases risk of some adverse developmental effects. Thus, it is important to characterize BPA levels, metabolic fate and sources of exposure in pregnant women.

**Methods:**

We used an improved liquid chromatography-tandem mass spectrometry (LC-MS/MS) analytic method to directly and simultaneously measure unconjugated BPA (uBPA), BPA glucuronide and BPA sulfate in the urine of a population of ethnically and racially diverse, and predominately low-income pregnant women (*n* = 112) in their second trimester. We also administered a questionnaire on dietary and non-dietary sources of exposure to BPA.

**Results:**

We found universal and high exposure to uBPA and its metabolites: median concentrations were 0.25, 4.67, and 0.31 μg/g creatinine for uBPA, BPA glucuronide, and BPA sulfate, respectively. The median Total BPA (uBPA + BPA in glucuronide and sulfate forms) level was more than twice that measured in U.S. pregnant women in NHANES 2005–2006, while 30 % of the women had Total BPA levels above the 95th percentile. On average, Total BPA consisted of 71 % BPA in glucuronide form, 15 % BPA in sulfate form and 14 % uBPA, however the proportion of BPA in sulfate form increased and the proportion of uBPA decreased with Total BPA levels. Occupational and non-occupational contact with paper receipts was positively associated with BPA in conjugated (glucuronidated + sulfated) form after adjustment for demographic characteristics. Recent consumption of foods and beverages likely to be contaminated with BPA was infrequent among participants and we did not observe any positive associations with BPA analyte levels.

**Conclusion:**

The high levels of BPA analytes found in our study population may be attributable to the low-income status of the majority of participants and/or our direct analytic method, which yields a more complete evaluation of BPA exposure. We observed near-universal exposure to BPA among pregnant women, as well as substantial variability in BPA metabolic clearance, raising additional concerns for effects on fetal development. Our results are consistent with studies showing thermal paper receipts to be an important source of exposure, point to the difficulty pregnant women have avoiding BPA exposure on an individual level, and therefore underscore the need for changes in BPA regulation and commerce.

**Electronic supplementary material:**

The online version of this article (doi:10.1186/s12940-016-0131-2) contains supplementary material, which is available to authorized users.

## Background

Bisphenol A (BPA) is a high production volume chemical with a broad range of applications, including resins that line food and beverage cans and fermentation tanks, polycarbonate plastics, medical devices, cigarette filters and toys [1–3]. BPA is also used as a developer in thermal paper (used for receipts and tickets) and in printing ink, and has been detected in both virgin and recycled paper goods, such as food and beverage contact paper (e.g., paper plates and napkins, cardboard boxes), newspapers and paper currency [4–10]. The State of California recently added BPA to its list of known developmental and reproductive toxicants: exposure to BPA, particularly during pregnancy, is of public health concern because BPA possesses a variety of hormone-like actions, and animal studies have shown that *in utero* exposure to BPA can alter the development of a wide range of organ systems (e.g., brain, prostate, mammary gland, lung, and reproductive tract) and accelerate the onset of puberty [11–16]. Furthermore, epidemiology studies have found associations between BPA exposure and adverse reproductive health effects, such as reduced semen quality, sperm DNA damage [17–19] and oocyte maturity and normal fertilization in women undergoing IVF [20].

BPA continues to be produced and used in consumer products in large quantities (averaging 8 lb per capita in the United States with a total of 2.3 billion pounds annually [3, 21]), resulting in ubiquitous human exposure: more than 90 % of the general and 96 % of the pregnant U.S. population have measurable levels of Total BPA (unconjugated BPA (uBPA) plus its primary conjugated metabolites, BPA glucuronide and BPA sulfate) in their urine [3, 22, 23]. Higher urinary levels of Total BPA have been measured in lower socioeconomic status U.S. women and children, and lower levels in U.S. Hispanic women and children, suggesting BPA exposure varies by race/ethnicity, sex and age [23–25].

Understanding sources of BPA exposure is instrumental for identifying populations at greatest risk and for devising strategies for reducing exposure. Studies measuring BPA in food and paper products have found levels as high as 730 ng/g in food (e.g., canned green beans) [26], 26.6 ug/g in food-contact paper [10] and 42.6 mg/g in thermal receipt paper [6]. Higher urinary concentrations of Total BPA have been associated with cashier-related occupations in pregnant women [27] and the general population [28, 29], with consumption of canned vegetables, hamburgers and soda in pregnant women [25], and with consumption of soda and meals prepared outside the home in the general U.S. population [30]. Smaller intervention studies of non-pregnant populations have also identified canned soup, plastic water bottles and handling thermal paper receipts as potential important sources of exposure [31–34]. Despite the importance of characterizing BPA exposure during pregnancy and the higher Total BPA levels reported in certain socioeconomic and racial/ethnic groups [22, 24], there has been little research on exposure levels and sources of exposure in ethnically and racially diverse and low-income groups of pregnant women [24].

Most biomonitoring studies to date have estimated prenatal exposure to Total BPA indirectly, using enzyme hydrolysis to cleave glucuronide and sulfate bonds with BPA, then quantifying uBPA [35]. Due to limitations in processing methodology (i.e., incomplete enzyme hydrolysis), this indirect methodology may underestimate exposure levels. In addition, emerging research points to the need for direct quantification of BPA glucuronide and BPA sulfate concentrations when determining the health risks posed by BPA, particularly to the developing fetus. First, though not observed in one rodent study [36], in vitro studies suggest that BPA glucuronide and BPA sulfate may be deconjugated by β-Glucuronidase (highly active in the placenta and fetal liver) or estrone sulfatase, respectively, thus leading to deconjugation-conjugation cycling of BPA [37, 38]. Second, recent science indicates that BPA glucuronide may also be biologically active [39], and this possibility for BPA sulfate has not been precluded.

In the current cross-sectional study, we employed a more sensitive and accurate analytic technique to directly and simultaneously measure uBPA, BPA glucuronide and BPA sulfate in a low-income, ethnically and racially diverse population of pregnant women. We also administered a comprehensive questionnaire to identify potential sources of exposure to BPA.

## Methods

### Study population and recruitment

We recruited pregnant women in their second trimester from the Women’s Options Center (WOC) at San Francisco General Hospital (SFGH) in San Francisco, California. The WOC is an academic-based outpatient clinic that performs pregnancy terminations and serves communities from Northern and Central California. We recruited pregnant women, 18 to 45 years of age, English- or Spanish-speaking, and pregnancy gestation between 13 and 24 weeks. Because our study objectives were to understand BPA exposure and exposure sources in healthy pregnancies, patients seeking a pregnancy termination due to fetal anomalies were excluded. We identified eligible study participants by reviewing the patient’s medical record only after she had 1) consulted with a trained counselor for an elective second trimester pregnancy termination procedure and 2) consented to the procedure as documentation of her intent to proceed with the elective pregnancy termination. Study protocols were approved by the University of California, San Francisco Committee on Human Research. We recruited a total of 185 participants between 2009 and 2011 and collected urine samples from 171. The integrity of 54 urine samples was potentially compromised due to a freezer malfunction, so they were not analyzed for BPA. Creatinine measurements were unavailable for 5 participants who were thus excluded, leaving a total of 112 pregnant women in the final study sample.

### BPA exposure questionnaire and demographic data

To evaluate dietary and non-dietary sources of exposure, we developed a BPA exposure questionnaire based on a literature review of human exposure assessment studies and measurements of BPA in various food and consumer products [2–4, 40]. The survey instrument included 197 questions regarding: consumption of foods and beverages that could be contaminated with BPA due to packaging or preparation; occupational and non-occupational contact with paper receipts; and knowledge and avoidance of BPA. Specifically, we asked about consumption of foods and beverages packaged in cans, cartons or paper, or served on paper plates, napkins or cups, as well as consumption of wine and beer. The survey captured both short-term (i.e., consumed “today”, “yesterday”) as well as long-term dietary consumption. Additional demographic and medical information collected through the questionnaire or medical record abstraction included: maternal age, gestational age of the fetus, body mass index (BMI), personal and combined household income, educational attainment, insurance status, food stamp assistance, smoking status during the past year and race/ethnicity. Seven percent of our study sample completed a self-administered questionnaire (*n* = 8) as part of the field testing of the questionnaire, while the remaining 93 % completed an interview-administered questionnaire (both modalities were supported by pictures and cue-cards). The BPA exposure questionnaire is available upon request from the corresponding author.

### Sample collection and laboratory analysis

A non-fasting spot urine sample was collected on the day prior to the medical procedure (before any medical interventions occurred) and after the participant completed the BPA exposure questionnaire. Urine samples were stored on ice until they were aliquoted into 5 ml polypropylene cryovials and then stored at −80 °C until analysis. Urinary creatinine levels were measured by the SFGH Clinical Chemistry Laboratory using the enzymatic creatinine method and the Siemens Advia 1800 autoanalyzer.

Direct analysis of BPA analytes was done by liquid chromatography-tandem mass spectrometry (LC-MS/MS). We measured uBPA, BPA glucuronide and BPA sulfate simultaneously using Agilent LC 1260-AB Sciex 5500 with electrospray ionization in the negative mode as previously described [41]. Each analyte was monitored by multiple reaction monitoring using two transitions and BPA-d16 as an internal standard: uBPA, 227.012 – 133.100 and 227.0 – 212.1; BPA glucuronide, 402.9 – 112.9 and 402.9 – 226.9; BPA sulfate, 306.9 – 227.0 and 306.9 – 212.1; and BPA-d16, 241.0 – 142.2 and 241.0 – 222.1. Each urine sample was thawed and centrifuged at 3000 rpm for 10 min before it was prepared for LC-MS/MS analysis by solid phase extraction (SPE) using Waters Oasis HLB cartridge (1 cm^3^). Each SPE cartridge was washed with 5 column volumes of methanol to eliminate its reported BPA contamination [41]. The cartridges were then activated with water before 500 μL of urine was loaded. The column was washed with 5 % methanol before each analyte was eluted by methanol. The methanol eluates were evaporated under a stream of nitrogen gas and reconstituted in 500 μL 10 % methanol for column injection. A 25 μL aliquot of the extract was used for each of the replicate injections of the sample. Chromatographic separation of the analytes was achieved by gradient elution using water with 0.05 % ammonium acetate (pH = 7.8) as mobile phase A and methanol with 0.05 % ammonium acetate (pH = 7.8) as mobile phase B. The elution gradient employed was: 0 – 0.5 min = 30 % B; 1 min = 75 % B; 4 min = 100 % B; 4 – 6 min = 100 % B; and 6.01 – 12 min = 30 % B. Quantification of each analyte was done by isotope dilution method using BPA-d16 as an internal standard. During method development, we also measured for BPA disulfate, but did not find significant levels in urine (data not shown).

We assessed the limit of detection (LOD) for each analyte by running a series of calibration standards (0.01 – 100 ng/mL), established as the lowest concentration of the analyte that gives a signal/noise (S/N) ratio of 3. We established the limit of quantification (LOQ) as the lowest concentration with S/N ratio of 10 while keeping the linear regression coefficient of the standard curve ≥ 0.95. The LODs for uBPA and BPA glucuronide were 0.05 ng/mL while the LOD for BPA sulfate was 0.025 ng/mL. The lower LOQ for all three analytes was 0.10 ng/mL. Method recoveries for each analyte were reproducibly high both within and between runs [41]. The range of recoveries obtained for uBPA, BPA glucuronide and BPA sulfate were 90.5 – 96.0 %, 87.5 – 91.0 % and 92 – 97.5 %, respectively. For each of the analytes, the recoveries were observed at a narrow range ensuring that analytical variability would not contribute significantly to measured levels of the analyte in the samples. The ranges of method precision within run and between runs established for uBPA, BPA glucuronide and BPA sulfate were 1.5 – 7.0 % coefficient of variation (CV), 3.0 – 9.5 % CV and 2.4 – 7.5 % CV, respectively.

We also tested all equipment and supplies used in the collection or storage of urine for their potential to contaminate urine specimens with BPA [41]. Specifically, we simulated sample collection, extraction and analytical run using synthetic human urine (UTAK Laboratories, Inc.). All processes and equipment were found to be free of BPA contamination (BPA < LOD in all field blank testing materials).

### Data analysis

We imputed samples below the LOD with LOD/√2 [23] and all analyte concentrations were adjusted for creatinine. In calculating Total BPA (the sum total concentration of all BPA analytes), it is necessary to account for the higher mass of BPA glucuronide and BPA sulfate compared to uBPA (due to the additional glucuronide and sulfate conjugate groups, respectively). Accordingly, we calculated two metrics – BPA in glucuronide form and BPA in sulfate form – that adjusted for the additional mass of the conjugate groups as follows: we multiplied the concentration of BPA glucuronide by the ratio of the molecular weight of BPA to that of BPA glucuronide (0.5645), and the concentration of BPA sulfate by the ratio of the molecular weight of BPA to that of BPA sulfate (0.7404). We summed uBPA, BPA in glucuronide form and BPA in sulfate form to calculate Total BPA and summed BPA in glucuronide form and BPA in sulfate form to calculate BPA in conjugated form. In addition to permitting a comparison of the relative levels of each BPA analyte within a sample, BPA in glucuronide form, BPA in sulfate form, BPA in conjugated form and Total BPA are the best metrics for comparing concentrations in this study to all previous studies that measured BPA glucuronide and/or BPA sulfate using enzyme hydrolysis methods.

We calculated geometric mean (GM), geometric standard deviation (GSD), median, inter-quartile range (IQR) and 95th percentile estimates for creatinine-adjusted and unadjusted BPA analytes. Distributions of uBPA, BPA in glucuronide form and BPA in sulfate form were highly right skewed. Therefore we used non-parametric tests to examine the correlation between BPA analytes as well as their univariate association with demographic characteristics and BPA exposure sources, and we used log-transformed BPA analyte levels in all multivariable analyses.

We aggregated data from the BPA exposure questionnaire to create 15 BPA exposure source variables that represent how recently (e.g., today, yesterday, not in past two days) a participant reported contact with each type of potential BPA exposure source (e.g., contact with paper receipts, consumption of canned and paper-packaged foods) in order to evaluate our hypothesis that recent exposure would be associated with higher levels of BPA analytes. We also generated long-term dietary exposure variables that represented the frequency and quantity of items typically consumed per week. Participants who answered “don’t know” to any question on the questionnaire were grouped with the “not-exposed” category if they comprised less than 5 % of the study sample. (The only question to which more than 5 % of participants answered “don’t know” pertained to combined household income; these 27 participants were grouped with those who chose “do not want to answer.”) In addition, only 4 participants reported consuming canned foods on the day of urine collection; therefore we grouped them with participants who reported eating canned foods “yesterday.” Because the identical set of BPA exposure source variables were associated with BPA in glucuronide form and BPA in sulfate form in a univariate context, we analyzed and report results for BPA in conjugated form.

For multivariable analyses of the association between log-transformed BPA analyte levels and BPA exposure sources, adjusting for demographic characteristics, we first identified demographic covariates associated with log-transformed uBPA and BPA in conjugated form via backwards stepwise regression (separate models were constructed for uBPA and BPA in conjugated form). Next, we estimated marginal geometric means (i.e., covariate-adjusted geometric means) of uBPA and BPA in conjugated form, with the largest subgroup of categorical variables serving as the baseline. Results were consistent whether we adjusted for creatinine by including creatinine as a covariate in the model or by using creatinine-adjusted log-transformed BPA analyte levels as the outcome variable. Therefore, we present the results of the latter approach so that crude and adjusted geometric means can be compared. Linear regression assumptions were checked for final models using normal-quantile and residual versus fitted plots and Shapiro-Wilk tests for normality of standardized residuals. We also performed two sensitivity analyses by refitting final models excluding: 1) 11 subjects with creatinine levels outside the World Health Organization (WHO) inclusionary range (30 – 300 mg/L) and 2) influential data points (|residuals| ≥ 2.5) for that model. We set the level of statistical significance at *p* < 0.10 for our smaller sample size. All statistical analyses were conducted in Stata (version 12). All BPA analyte levels presented are creatinine-adjusted values unless otherwise specified (creatinine measurements ranged from 15.42 to 357.07 mg/dL).

## Results

**Fig. 1:**
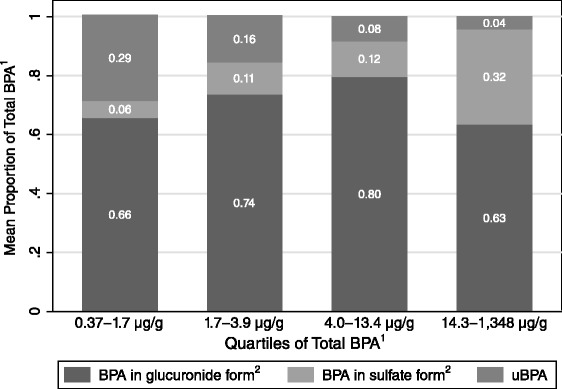
Composition of Urinary Total BPA in Second Trimester Pregnant Women, by quartiles of Creatinine-Adjusted Total BPA Levels, Northern and Central California, 2009–2011 (*n* = 112). Notes: 1. Total BPA = BPA + BPA in glucuronide form + BPA in sulfate form. 2. BPA in glucuronide form = BPA glucuronide*0.5614. BPA in sulfate form = BPA sulfate*0.7404. The factors 0.5614 and 0.7404 are the ratios of the molecular weight of BPA to the molecular weights of BPA glucuronide and BPA sulfate, respectively

All study participants had detectable concentrations of uBPA, BPA glucuronide or BPA sulfate in their urine (Table 1). Total BPA levels were right skewed – 38 (>30 %) participants had Total BPA levels ≥ 10 μg/g creatinine, 20 (>17 %) had Total BPA levels ≥ 50 μg/g creatinine and 15 (>13 %) had Total BPA levels ≥ 100 μg/g creatinine – and highly variable, with a more than three orders of magnitude range across study participants (CV = 3.4). Concentrations of BPA in glucuronide form were higher than other BPA forms in 85 % of urine samples, whereas BPA in sulfate form was higher than other BPA forms in 5 % of participants who also had higher Total BPA levels (GM = 5.7 vs. 19.4 ng/g, Kruskall Wallis *p* = 0.07). On average, Total BPA was comprised of 71 % BPA in glucuronide form (standard deviation (SD) 23 %, median 77 %), 15 % BPA in sulfate form (SD 18 %, median 8 %), and 14 % BPA (SD 22 %, median 4 %). However, the proportion of Total BPA in sulfate form varied by race/ethnicity (whites 16 %, Latinas 12 %, Asian/Pacific Islanders 10 % and Blacks 4 %, Kruskall Wallis *p* = 0.0079) and increased with Total BPA levels (Spearman rho (ρ) = 0.56, *p* < 0.0001), such that BPA in sulfate form was 32 % of Total BPA in women in the top quartile of Total BPA levels (Fig. 1). As Total BPA levels increased, the proportion of BPA in glucuronide form remained unchanged (*p* = 0.62) and the proportion of BPA decreased (ρ = -0.68, *p* < 0.0001) (Fig. 1). Consistent with these findings, we found that levels of BPA in glucuronide and sulfate form were highly correlated with each other (ρ = 0.83, *p* < 0.0001) but not with uBPA (Additional file 1), while women with Total BPA levels above the GM had higher levels of BPA in glucuronide and sulfate forms (*p* < 0.0001) but similar levels of uBPA compared to women with Total BPA levels below the GM (Additional file 2).

**Table 1:** Urinary levels of BPA analytes in second trimester pregnant women, Northern and Central California, 2009–2011 (n = 112)

			Percentile					
	n (%) >LOD^a^	GM (GSD)	5^th^	25^th^	50^th^	75^th^	95^th^	Range
Creatinine-adjusted (μg/g)								
Total BPA^b^	112 (100)	6.16 (1.77)	0.61	1.69	3.97	13.87	196.65	0.37 - 1,347.50
BPA	98 (88)	0.21 (1.39)	<LOD	0.08	0.25	0.55	1.61	<LOD - 16.96
BPA glucuronide	111 (99)	6.77 (1.90)	0.60	2.16	4.67	15.31	249.76	<LOD - 2,019.33
BPA in glucuronide form^c^	111 (99)	3.82 (1.90)	0.34	1.22	2.64	8.64	140.99	<LOD - 1,139.91
BPA sulfate	84 (75)	0.62 (2.73)	<LOD	0.05	0.42	3.54	80.35	<LOD - 279.77
BPA in sulfate form^c^	84 (75)	0.46 (2.73)	<LOD	0.04	0.31	2.62	59.49	<LOD - 207.14
Unadjusted (ng/mL)								
Total BPA^b^	112 (100)	7.69 (1.74)	0.88	2.28	4.61	19.49	250.06	0.35 - 539.63
BPA	98 (88)	0.26 (1.20)	<LOD	0.10	0.29	0.66	1.51	<LOD - 4.41
BPA glucuronide	111 (99)	8.45 (1.93)	0.57	2.49	6.74	21.08	359.41	<LOD - 683.42
BPA in glucuronide form^c^	111 (99)	4.77 (1.93)	0.32	1.41	3.80	11.90	202.89	<LOD - 385.79
BPA sulfate	84 (75)	0.77 (2.71)	0.04	0.04	0.49	5.97	109.24	0.04 - 271.16
BPA in sulfate form^c^	84 (75)	0.57 (2.71)	0.03	0.03	0.37	4.42	80.88	0.03 - 200.77

*GM* geometric mean, *GSD* geometric standard deviation, *LOD* limit of detection

^a^LOD = 0.05 ng/mL for BPA and BPA glucuronide and LOD = 0.025 ng/mL for BPA sulfate

^b^Total BPA = BPA + BPA in glucuronide form + BPA in sulfate form

^c^BPA in glucuronide form = BPA glucuronide*0.5614. BPA in sulfate form = BPA sulfate*0.7404. The factors 0.5614 and 0.7404 are the ratios of the molecular weight of BPA to the molecular weights of BPA glucuronide and BPA sulfate, respectively

Our study sample was ethnically and racially diverse and primarily low income (Table 2). African Americans were the most represented and Latina and white women each comprised approximately 25 % of the sample. Sixty-five percent of participants had a 12th grade or lower education, 41 % reported a combined annual household income of less than $20,000 (only 12 subjects reported household incomes of $40,000 or more), and nearly all women received public health insurance. BPA in conjugated form was positively associated with education and nulliparity in a univariate context, and with education and race/ethnicity in a multivariable context. uBPA was positively associated with education and negatively associated with BMI in both a univariate and multivariable context.

**Table 2:** Characteristics and creatinine-adjusted urinary BPA concentrations of second trimester pregnant women from Northern and Central California, 2009–2011 (n=112)

	n (%)	BPA in Conjugated Form^a^ (μg/g) GM (95 % CI)	BPA (μg/g) GM (95 % CI)
Ethnicity			
Latina	26 (23)	6.52 (2.94, 14.50)	0.17 (0.10, 0.30)
Black	41 (37)	2.71 (1.68, 4.38)	0.23 (0.14, 0.37)
White	27 (24)	8.60 (3.39, 21.82)	0.27 (0.16, 0.46)
Asian/PI/Unknown	18 (16)	4.97 (1.72, 14.33)	0.15 (0.08, 0.28)
Smoking Status^b^			
Non-smoker	64 (60)	5.48 (3.37, 8.90)	0.20 (0.14, 0.28)
Current smoker	43 (40)	4.02 (2.10, 7.69)	0.21 (0.14, 0.30)
Education^b^			
11^th^ Grade and below	19 (18)	2.26 (0.96, 5.36)^**^	0.11 (0.06, 0.22)^*^
12^th^ Grade/GED	51 (47)	3.74 (2.20, 6.37)	0.23 (0.15, 0.35)
College or above	38 (35)	10.45 (5.46, 20.00)	0.25 (0.17, 0.38)
Food-stamp Status			
No	52 (46)	6.46 (3.82, 10.93)	0.23 (0.15, 0.34)
Yes	60 (54)	3.77 (2.22, 6.39)	0.20 (0.14, 0.28)
Parity^b^			
Nulliparous	39 (35)	8.74 (4.39, 17.42)^**^	0.23 (0.15, 0.35)
Parity ≥1	73 (65)	3.53 (2.30, 5.42)	0.20 (0.14, 0.28)
Combined Annual Household Income ($)			
< 20,000	46 (41)	4.03 (2.35, 6.89)	0.20 (0.13, 0.30)
20,000 – 40,000	11 (10)	3.77 (0.87, 16.34)	0.21 (0.09, 0.51)
> 40,000	13 (12)	16.35 (4.02, 66.45)	0.18 (0.07, 0.43)
Refused/Don’t know	42 (37)	4.34 (2.37, 7.94)	0.23 (0.15, 0.36)
Medi-Cal Insurance^b^			
No	20 (18)	5.01 (2.04, 12.28)	0.15 (0.08, 0.29)
Yes	90 (82)	4.69 (3.07, 7.16)	0.23 (0.17, 0.31)
	Median (Range)	Spearman ρ	Spearman ρ
Age	25.4 (18.0 - 45.0)	0.00	−0.05
BMI	28.6 (17.2 - 65.8)	−0.02	−0.27^***^
Gestational Age, weeks	20.0 (13.3 - 24.0)	0.01	0.01
Urine Collection time	12:50 pm (10:00 AM - 4:50 PM)	0.04	−0.12

^a^BPA in Conjugated form = BPA in glucuronide form + BPA in sulfate form. BPA in glucuronide form = BPA glucuronide*0.5614. BPA in sulfate form = BPA sulfate*0.7404. The factors 0.5614 and 0.7404 are the ratios of the molecular weight of BPA to the molecular weights of BPA glucuronide and BPA sulfate, respectively

^b^Data missing on: smoking status (*n* = 5), education (*n* = 4), parity (*n* = 3), and Medi-Cal insurance status (*n* = 2)

* = *p* <0.1 and ** = *p* <0.05 for Kruskal-Wallis test

^***^ = *p* <0.05 for Spearman Correlation test

About half of participants reported touching or holding paper receipts on the day of urine collection and 21 % held receipt-related occupations (Table 3). In contrast, only a small percentage (0–26 %) reported consuming one or more of the five potential dietary sources of BPA on the day of urine collection, which is consistent with the relatively low frequency (median 0.5 – 4.75 times per week) with which participants reported consuming these foods and beverages on a regular basis (Additional file 3).

**Table 3:** Crude and adjusted BPA analyte levels, by sources of exposure, in second trimester pregnant women from Northern and Central California, 2009–2011 (n=112)

		BPA in Conjugated Form^a^ (μg/g creatinine)		BPA (μg/g creatinine)	
	n (%)	GM (95 % CI)	MGM (95 % CI)	GM (95 % CI)	MGM (95 % CI)
Job involves handling paper receipts					
No^d^	89 (79)	4.11 (2.70, 6.25)	4.21 (2.83, 6.26)	0.22 (0.16, 0.29)	0.21 (0.16, 0.27)
Yes	23 (21)	9.13 (4.10, 20.37)^**^	8.98 (4.07, 19.83)^***^	0.19 (0.11, 0.32)	0.18 (0.11, 0.32)
Touched or held paper receipts^b^					
Not in past 2 days	18 (16)	2.85 (1.03, 7.89)	3.78 (1.53, 9.35)	0.24 (0.11, 0.49)	0.22 (0.12, 0.41)
Yesterday	37 (33)	3.57 (2.00, 6.35)	3.40 (1.84, 6.27)^***^	0.16 (0.10, 0.27)	0.19 (0.12, 0.29)
Today^d^	57 (51)	6.98 (4.02, 12.09)	6.87 (4.16, 11.35)	0.24 (0.17, 0.33)	0.21 (0.15, 0.30)
Drank canned drinks					
Not in past 2 days^d^	57 (51)	6.32 (3.83, 10.41)	6.24 (3.81, 10.24)	0.21 (0.14, 0.31)	0.20 (0.14, 0.28)
Yesterday	44 (39)	3.65 (1.92, 6.95)	3.44 (1.94, 6.10)	0.22 (0.15, 0.34)	0.22 (0.15, 0.32)
Today	11 (10)	3.77 (1.05, 13.55)	5.73 (1.77, 18.59)	0.16 (0.06, 0.45)	0.18 (0.08, 0.40)
Drank cartoned drinks					
Not in past 2 days^d^	79 (71)	5.75 (3.58, 9.22)	5.81 (3.80, 8.86)	0.22 (0.17, 0.30)	0.23 (0.17, 0.30)
Yesterday	22 (20)	3.72 (1.78, 7.78)	3.73 (1.61, 8.64)	0.23 (0.12, 0.44)	0.16 (0.09, 0.30)
Today	11 (10)	2.38 (0.90, 6.33)	2.32 (0.68, 7.95)	0.11 (0.03, 0.32)	0.13 (0.06, 0.28)
Drank from paper cup					
Not in past 2 days^d^	55 (49)	5.55 (3.19, 9.66)	5.53 (3.35, 9.14)	0.22 (0.16, 0.32)	0.25 (0.18, 0.35)
Yesterday	28 (25)	3.18 (1.61, 6.28)	3.23 (1.53, 6.79)	0.15 (0.09, 0.28)	0.14 (0.08, 0.23)^***^
Today	29 (26)	5.61 (2.56, 12.29)	5.80 (2.82, 11.93)	0.24 (0.14, 0.42)	0.20 (0.12, 0.32)
Ate canned foods^c^					
Not in past 2 days^d^	75 (67)	5.60 (3.52, 8.92)	5.58 (3.61, 8.62)	0.18 (0.13, 0.24)	0.18 (0.14, 0.25)
Yesterday or today	37 (33)	3.60 (1.91, 6.76)	3.78 (2.01, 7.11)	0.30 (0.19, 0.46)	0.25 (0.16, 0.39)
Ate food served or packaged in paper or cardboard					
Not in past 2 days^d^	44 (39)	5.77 (2.99, 11.15)	5.87 (3.30, 10.45)	0.25 (0.17, 0.37)	0.27 (0.18, 0.40)
Yesterday	38 (34)	3.45 (1.94, 6.14)	3.57 (1.92, 6.63)	0.16 (0.10, 0.25)	0.15 (0.10, 0.23)^****^
Today	30 (27)	5.75 (2.74, 12.04)	5.62 (2.85, 11.09)	0.23 (0.13, 0.41)	0.19 (0.12, 0.31)
Purchases >1/2 of all meals^b^					
No^d^	69 (64)	4.37 (2.66, 7.18)	4.42 (2.81, 6.95)	0.26 (0.18, 0.36)	0.24 (0.18, 0.33)
Yes	38 (36)	5.37 (2.87, 10.04)	5.55 (2.94, 10.47)	0.15 (0.09, 0.23)^*^	0.15 (0.10, 0.23)^***^
Consumes wine^b^					
No^d^	68 (62)	5.90 (3.72, 9.37)	6.39 (4.04, 10.12)	0.20 (0.14, 0.27)	0.20 (0.14, 0.27)
Yes	42 (38)	3.20 (1.71, 5.99)^**^	2.95 (1.65, 5.30)^****^	0.24 (0.15, 0.39)	0.22 (0.15, 0.33)
Consumes beer					
No^d^	75 (67)	4.37 (2.69, 7.11)	4.84 (3.10, 7.55)	0.21 (0.15, 0.29)	0.21 (0.16, 0.29)
Yes	37 (33)	5.95 (3.39, 10.45)	5.06 (2.72, 9.42)	0.21 (0.14, 0.33)	0.19 (0.12, 0.28)
Eats food stored in clear, shatterproof plastic containers^b^					
No	38 (35)	4.93 (2.67, 9.10)	5.45 (2.98, 9.97)	0.26 (0.15, 0.45)	0.24 (0.16, 0.37)
Yes^d^	70 (65)	4.68 (2.90, 7.56)	4.55 (2.91, 7.13)	0.19 (0.14, 0.25)	0.18 (0.14, 0.25)
Uses food processor^b^					
No^d^	92 (84)	4.43 (2.94, 6.67)	4.23 (2.89, 6.20)	0.23 (0.17, 0.31)	0.21 (0.16, 0.28)
Yes	17 (16)	8.44 (3.19, 22.33)	12.81 (5.03, 32.60)^****^	0.14 (0.08, 0.26)	0.18 (0.09, 0.34)
Uses blender with plastic pitcher^b^					
No^d^	82 (76)	5.25 (3.35, 8.21)	5.35 (3.53, 8.10)	0.22 (0.16, 0.30)	0.21 (0.16, 0.28)
Yes	26 (24)	3.93 (1.91, 8.08)	4.05 (1.95, 8.41)	0.19 (0.11, 0.34)	0.20 (0.12, 0.32)
Uses kitchen appliance with plastic bowl/pitcher^b^					
No^d^	58 (54)	5.58 (3.31, 9.41)	5.63 (3.47, 9.13)	0.23 (0.16, 0.33)	0.20 (0.15, 0.29)
Yes	49 (46)	3.92 (2.24, 6.84)	4.02 (2.35, 6.88)	0.19 (0.13, 0.29)	0.20 (0.14, 0.29)
Knows or heard about BPA^b^					
No^d^	65 (60)	3.94 (2.54, 6.10)	4.32 (2.69, 6.93)	0.20 (0.14, 0.28)	0.19 (0.14, 0.27)
Yes	44 (40)	6.55 (3.33, 12.89)	5.95 (3.37, 10.50)	0.23 (0.15, 0.34)	0.21 (0.14, 0.31)
Takes action to avoid BPA					
None^d^	81 (72)	5.16 (3.28, 8.12)	5.31 (3.48, 8.10)	0.20 (0.15, 0.28)	0.20 (0.15, 0.27)
Avoids BPA or purchases	14 (12)				
BPA-free labeled products		2.16 (0.85, 5.48)	2.48 (0.90, 6.85)	0.30 (0.15, 0.62)	0.25 (0.12, 0.51)
Avoids BPA and purchases	17 (15)				
BPA-free labeled products		6.93 (2.67, 17.99)	6.09 (2.46, 15.08)	0.18 (0.08, 0.40)	0.18 (0.10, 0.34)

*GM* geometric mean, *MGM* marginal geometric mean

^a^BPA in conjugated form = BPA in glucuronide form + BPA in sulfate form. BPA in glucuronide form = BPA glucuronide*0.5614. BPA in sulfate form = BPA sulfate*0.7404. The factors 0.5614 and 0.7404 are the ratios of the molecular weight of BPA to the molecular weights of BPA glucuronide and BPA sulfate, respectively

^b^Data missing on: touch receipts (*n* = 5), purchased meals (*n* = 5), consume wine (*n* = 2), store food in plastic food container (*n* = 4), food processor (*n* = 3), blender with plastic pitcher (*n* = 4), kitchen appliance with plastic bowl/pitcher (*n* = 5), and knowledge of BPA (*n* = 3)

^c^4 participants who reported eating canned foods were grouped with “Ate yesterday” category

^d^ = baseline group

^*^ *p* <0.1 and ^**^ = *p* <0.05 for Kruskal-Wallis test

^***^ **=** *p* <0.1 and ^****^ = *p* <0.05 for multivariable linear regression, controlling for maternal education and race/ethnicity (for BPA in conjugated form outcome) and education and BMI (for BPA outcome)

In the multivariable analysis, BPA in conjugated form was significantly associated with employment in a job that involves dermal contact with paper receipts: After adjustment for education and race/ethnicity, participants who reported handling credit card or store receipts as part of their job had more than 2-fold higher GM concentrations of BPA in conjugated form (8.98 μg/g) compared to those who did not (4.21 μg/g, *p* < 0.10). Consistent with this finding, we observed a significant positive trend between recent non-occupational contact with paper receipts and levels of BPA in conjugated form (non-parametric test for trend, *p* < 0.05) and approximately 2-fold higher GM concentrations of BPA in conjugated form in women reporting holding or touching paper receipts the day of urine collection compared to those who did so on the day before (*p* = 0.08); however, this latter finding was no longer statistically significant when influential points were removed from the regression model. In a separate analysis to explore these findings, we observed a significant positive correlation between BPA in conjugated form and the number of times a participant reported touching receipts in the past two days (ρ = 0.29, *p* = 0.002). Regarding other potential BPA exposure sources, with the exception of food processor use (positive association) and wine consumption (negative association), we did not observe any statically significant associations with BPA in conjugated form in a multivariable context.

Levels of uBPA were positively associated with education and negatively with BMI in our multivariable analysis. However, uBPA was not associated with any BPA exposure source variables in a manner consistent with our hypothesis of recent exposure leading to higher urinary uBPA analyte levels (Table 3). Levels of uBPA were lower in women who purchased more than half of their meals in both univariate and multivariable analyses, however this association was not significant in the sensitivity analysis models (*p* = 0.6 and *p* = 0.35 when influential points and creatinine levels outside WHO range were excluded, respectively).

Women who reported knowledge of BPA or taking action to avoid BPA had similar levels of uBPA and BPA in conjugated form as women who did not (Table 3). Lastly, long-term dietary exposure scores were not associated with concentrations of either uBPA or BPA in conjugated form (Additional file 4).

To confirm the high levels of BPA glucuronide we observed, we sent six urine samples with high (*n* = 3) and low (*n* = 3) levels of BPA glucuronide to the University of Missouri at Columbia (blinded to our results) for direct measurement via LC-MS/MS [42]. The University of Missouri results were 100 % consistent with ours in terms of distinguishing samples with high and low BPA glucuronide levels and in terms of comparative concentrations (Table 4).

**Table 4:** University of Missouri confirmation of urinary BPA glucuronide levels in second trimester pregnant women from Northern and Central California, 2009–2011 (n=112)

	Direct measurement of BPA glucuronide (ng/mL)	
Sample	University of Missouri	UCSF
A	2.96	0.80
B	6.82	5.95
C	14.25	10.22
D	221.49	439.56
E	332.73	463.70
F	1069.87	671.67

## Discussion

**Fig. 2:**
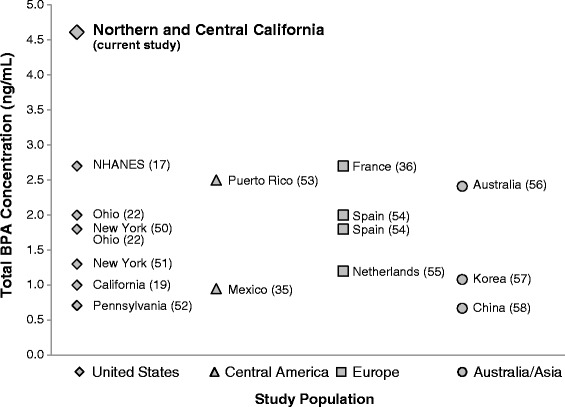
Median Urinary Total BPA levels (ng/mL) in studies^1^ of pregnant women. 1. Samples sizes: Current study, *n* = 112; United States (NHANES) [22], *n* = 86; Massachusetts [62], *n* = 84; Ohio [63], *n* = 244; New York [64], *n* = 198; Ohio [27], *n* = 339; New York [65], *n* = 404; California [25], *n* = 491; Canada [66], *n* = 1,876; Pennsylvania [67], *n* = 10; Puerto Rico [68], *n* = 105; Mexico [43], *n* = 60; France [44]), *n* = 287; Spain [69], *n* = 120; Netherlands [70], *n* = 100; Australia [71], *n* = 26; Korea [72], *n* = 757; China [73], *n* = 567.2. Total BPA = BPA + BPA in glucuronide form + BPA in sulfate form. 3.BPA in glucuronide form = BPA glucuronide*0.5614. BPA in sulfate form = BPA sulfate*0.7404. The factors 0.5614 and 0.7404 are the ratios of the molecular weight of BPA to the molecular weights of BPA glucuronide and BPA sulfate, respectively

We applied an analytic method to directly, and therefore more specifically and accurately, measure urinary uBPA, BPA glucuronide, and BPA sulfate, and found universal and unprecedentedly high levels in our racially/ethnically diverse sample of primarily low-income women in their second trimester of pregnancy. The median concentration of Total BPA in our study (4.61 ng/mL) was twice the levels reported in pregnant women in the United States and other countries (range 0.7 – 2.7 ng/mL) (Fig. 2) [22, 25, 27, 43, 44]. Moreover, 30 % of our participants had creatinine-adjusted Total BPA levels that were higher than the 95th percentile reported among U.S. pregnant women in the 2005–2006 NHANES [22]. We also detected uBPA in greater than 85 % of study participants and observed high variability in the relative proportions of uBPA and BPA metabolites detected in urine samples.

The high values of BPA glucuronide in some of our subjects were confirmed in split samples analyzed by the University of Missouri, which was blinded to our results. Our results were 100 % consistent in that we both distinguished high and low BPA glucuronide levels as wel as the ranking of the BPA glucuronide levels in the samples. The differences in absolute values we observed between the two laboratories’ measurements may be due to the use of different sample extraction methods and/or sources of reference and internal standards. Although both laboratories used solid phase extraction, we used different columns (our laboratory used Waters Oasis HLB SPE cartridge while the University of Missouri used Thermo Hypersep C18 SPE cartridge). Likewise, the sources of reference and internal standards differ between the two laboratories. These differences could have resulted in differences in the accuracy, precision and recovery of the methods in each laboratory. Further cross-validation is needed to identify the exact sources of the differences in the absolute values.

Although women in the highest annual household income bracket had the highest geometric mean levels of BPA in conjugated form, our study population as a whole was predominantly low income (50 % reporting had annual household incomes < $40,000). Low income has been associated with higher Total BPA levels in previous studies, possibly due to differential sources of exposure [23, 24], and thus may explain, in part, the high levels of BPA analytes we observed. None of the other BPA exposure sources we investigated were significantly associated with having high (i.e., >50 ng/mL) levels of Total BPA (data not shown).

Our improved LC-MS/MS analytic methods could also explain the higher Total BPA levels we observed. We used LC-MS/MS to directly measure uBPA, BPA glucuronide, and BPA sulfate, whereas most previous studies used enzyme hydrolysis followed by HPLC-MS/MS [42]. The use of enzyme hydrolysis may underestimate BPA analyte levels if these reactions are incomplete or do not otherwise function as predicted, and it is plausible that this underestimation would be greatest at the highest concentrations of glucuronidated or sulfated BPA. The results of our preliminary exploration of this hypothesis indicate that the indirect method of quantification may underestimate samples with very high levels of BPA glucuronide by up to a factor of four and that, although BPA glucuronide is fully deconjugated by commercial glucuronidases, only a small fraction of it is converted to uBPA (which indirect methods use as a surrogate measure for conjugated BPA). These findings suggest that human exposure to BPA has been previously underestimated. Further studies to determine the major product formed from the deconjugation of BPA glucuronide by commercial beta-gluduronidases are underway.

Although direct measurements of BPA have been made in the general population [45], to our knowledge, this is the first study to report direct measurements of uBPA in urine of pregnant women. Our detection of uBPA in 87 % of urine samples challenges prior assumptions that internal exposure to uBPA is limited due to rapid first-pass glucuronide metabolism [46, 47]. Whether the presence of uBPA in urine is due to incomplete metabolic clearance, dermal or inhalation exposure (which do not undergo first-pass metabolism), the deconjugation of BPA glucuronide or BPA sulfate by β-Glucuronidases or estrone sulfatase, respectively, the release of uBPA stored in tissues, other unidentified sources, or combinations thereof cannot be ascertained from our data. Nevertheless, our findings underscore the importance of obtaining a better understanding of the routes of human exposure to BPA as well as human metabolic capacity and pathways for BPA, as all of these factors have important implications for risk assessment.

We observed large variability in the relative proportions of uBPA, BPA in glucuronide form and BPA in sulfate form across study participants, with approximately 5 % of the population having higher levels of BPA in sulfate form than BPA in glucuronide form. This could be due, in part, to increased activity in secondary metabolic pathways in response to the saturation of glucuronidation capacity (as seen with immature glucuronidation capacity in the fetus [48–50]): the lack of association between Total BPA and BPA in glucurondiated form, coupled with the positive correlation between Total BPA and BPA in sulfated form seen in our population, suggest that glucuronidases are either saturated or inhibited at high BPA load, thus diverting metabolism to the sulfation pathway. An additional factor that may influence BPA metabolism is polymorphisms in genes encoding glucuronidation and sulfation enzymes. Specifically, several of the primary enzymes involved in BPA glucuronidation and sulfation have functional polymorphisms that yield variants with significantly lower or higher metabolic activity [51–53]. For example, SULT 1A1, a common sulfonation enzyme of small planar phenols like BPA, is polymorphic. Its *1 allele is known to significantly sulfonate faster than its *2 and *3 alleles [54]. Our findings highlight the importance of quantifying the complete suite of BPA conjugates and the need for additional research on factors that influence their concentrations.

We found that women with occupations that required handling of receipts and women who recently touched paper receipts had higher levels of BPA in conjugated form. These results are consistent with the findings of Braun et al., who found a positive association between occupation as a cashier and Total BPA levels in pregnant women [27], as well as other studies of the general US population [28, 29], and Hormann et al., who demonstrated dermal and oral absorption of uBPA resulting from holding thermal receipt paper followed by consumption of hand-held food [55]. Several studies have shown that thermal paper (including that used for cash register receipts) contains miligrams per gram levels of BPA [5–8, 56–58] and that BPA can be transferred from receipts to skin and persist there even after hand-washing [56]. Our results support the importance of this source of exposure to BPA and also suggest that receipt-related exposure to BPA is not limited to those who are occupationally exposed.

Another notable finding is that participants who reported knowledge of or action to avoid BPA did not have lower levels of BPA analytes in their urine. This is consistent with a recent randomized dietary trial that concluded that education and written guidelines are insufficient to reduce BPA exposure and only federal or industrial actions can completely eliminate exposure to BPA via the food supply [59] and with the lack of awareness of thermal paper as a source of BPA exposure during the time that our study was conducted.

Unlike other U.S. studies that report the lowest Total BPA levels in Hispanic pregnant women, we found the lowest levels of Total BPA in African Americans. Our findings also did not confirm the previously reported associations between Total BPA and SES, age, smoking, or consumption of canned soup and vegetables or purchased foods [23–25, 27, 30, 33, 60, 61]. However, this could be due to lack of variability in our sample with respect to these attributes. Our study participants are predominately low income and represent a narrow age range, and although the average canned food and beverage consumption in our sample was 5.6 and 10.2 times/week, respectively, only four participants reported consuming canned foods and 11 reported drinking canned beverages on the day of urine collection. Thus, it is likely that we lack sufficient sample size to evaluate these sources of exposure. Lastly, we relied on self-reported measures of smoking while other studies measured biomarkers of environmental tobacco smoke [27, 61], which may account for our null findings.

Our study has several limitations. Total BPA levels have been found to fluctuate during different stages of pregnancy [25] and throughout the day [27]. We collected a single spot urine sample and therefore could not account for this intra-individual variability in our evaluation of exposure sources. However, we did not observe any association between any BPA analyte (creatinine-adjusted or not) and time of urine collection previously reported in other studies of pregnant women [25, 27], possibly because urine was collected within a narrow time range (Table 2). Another weakness is our modest sample size and large number of investigated exposure sources. False positives may have arisen from multiple comparisons, and our results warrant confirmation in future studies.

## Conclusion

Our study provides novel data on uBPA, BPA glucuronide and BPA sulfate in urine of a low-income and ethnically and racially diverse sample of pregnant women, a sizeable subset of which has substantially elevated levels. We observed near-universal exposure to biologically active BPA among pregnant women as well as substantial variability in BPA metabolic clearance, raising additional concerns for effects on fetal development. Our results also indicate that dermal contact with thermal paper receipts is an important source of exposure than could be mitigated. Lastly, our work points to the difficulty pregnant women have avoiding BPA exposure on an individual level and therefore underscores the need for changes that focus on the use and sources of BPA in commerce.

## Additional files

Additional file 1: Spearman’s correlation between creatinine-adjusted BPA analytes in Second Trimester Pregnant Women (PDF 271 kb) Additional file 2: Urinary BPA analyte concentrations by log-transformed, creatinine-adjusted levels of Total BPA in Second Trimester Pregnant Women (PDF 191 kb) Additional file 3: Long-term dietary exposure to potential sources of BPA, and correlation with urinary BPA in Conjugated Form1 and uBPA in Second Trimester Pregnant Women (DOCX 21 kb) Additional file 4: University of Missouri Laboratory Confirmation of BPA glucuronide: Laboratory Methods (DOCX 25 kb)

## Abbreviations

BMI: body mass index
BPA: Bisphenol A
cc: cubic centimeter
CV: coefficient of variation
GM: geometric mean
GSD: geometric standard deviation
IQR: inter-quartile range
LC-MS/MS: liquid chromatography-tandem mass spectrometry
LOD: limit of detection
LOQ: limit of quantification
ng/mL: nanogram per milliliter
NHANES: National Health and Nutrition Examination Survey
rpm: rotations per minute
SD: standard deviation
SFGH: San Francisco General Hospital
SPE: solid phase extraction
uBPA: unconjugated BPA
WHO: World Health Organization
WOC: Women’s Options Center
μg/g: micrograms per gram
μL: microliter
ρ: Spearman rho
