# Supplementary material for: Direct measurement of Bisphenol A (BPA), BPA glucuronide and BPA sulfate in a diverse and low-income population of pregnant women reveals high exposure, with potential implications for previous exposure estimates: a cross-sectional study
Source: Environ Health. 2016 Apr 12;15:50. doi: 10.1186/s12940-016-0131-2 (PMC4828888; doi:10.1186/s12940-016-0131-2)
Supplement: Additional file 2: — Urinary BPA analyte concentrations by log-transformed, creatinine-adjusted levels of Total BPA in Second Trimester Pregnant Women (PDF 191 kb) [file 12940_2016_131_MOESM2_ESM.pdf]

Direct measurement of Bisphenol A (BPA), BPA glucuronide and BPA sulfate in a diverse and low-income population of pregnant women reveals high exposure, with potential implications for previous exposure estimates: a cross-sectional study.

Roy R. Gerona, Janet Pan, Ami R. Zota, Jackie M. Schwartz, Matthew Friesen, Julia A. Taylor, Patricia A. Hunt, Tracey J. Woodruff

Additional File 2

Urinary BPA analyte concentrations by log-transformed, creatinine-adjusted levels of Total BPA in Second Trimester Pregnant Women, Northern and Central California, 2009-2011 (n=112)

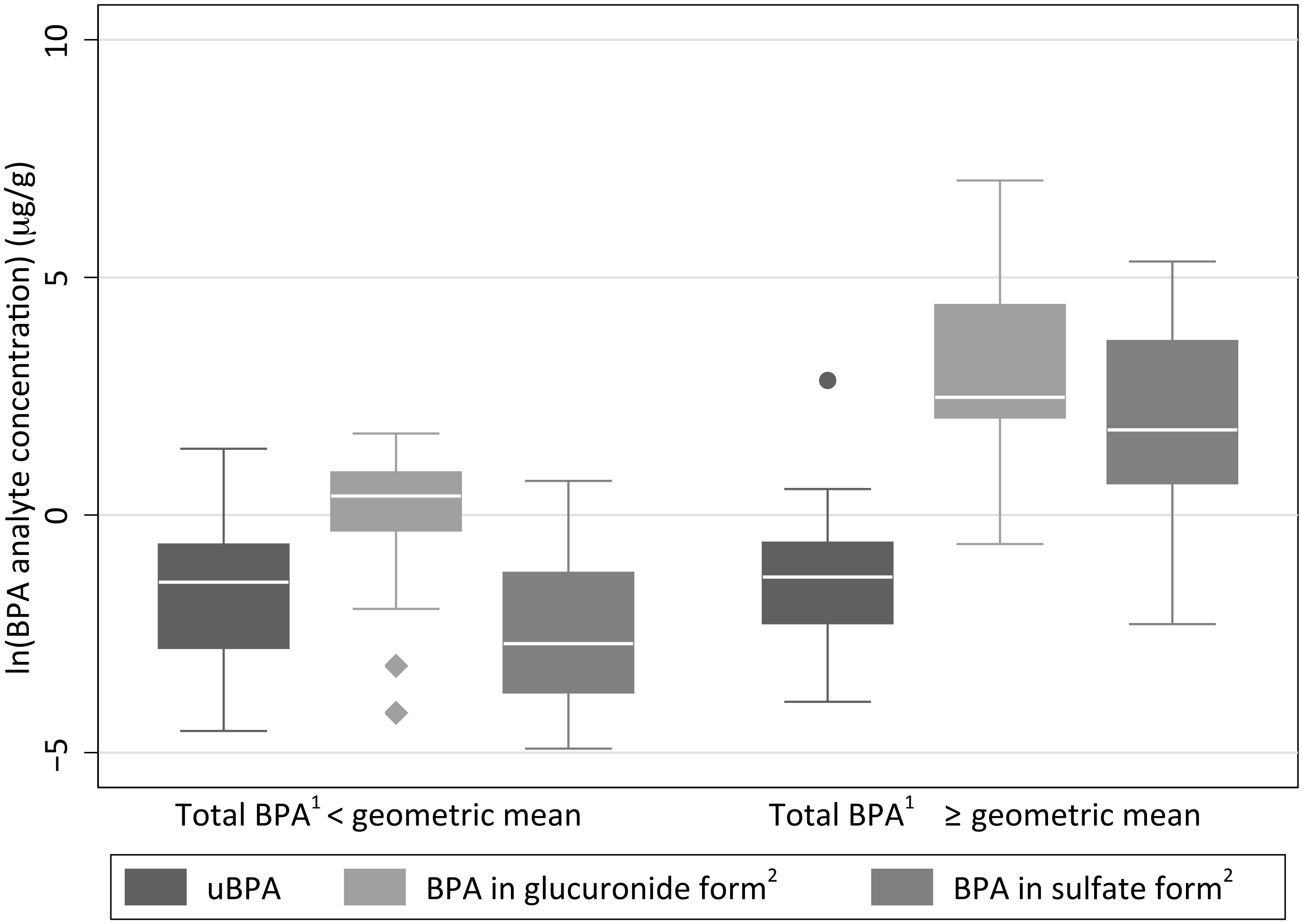

1. BPA in glucuronide form=BPA glucuronide*0.5614. BPA in sulfate form=BPA sulfate*0.7404. The factors 0.5614 and 0.7404 are the ratios of the molecular weight of BPA to the molecular weights of BPA glucuronide and BPA sulfate, respectively.
2. BPA analyte concentrations log-transformed and creatinine-adjusted
