# Supplementary material for: Direct measurement of Bisphenol A (BPA), BPA glucuronide and BPA sulfate in a diverse and low-income population of pregnant women reveals high exposure, with potential implications for previous exposure estimates: a cross-sectional study
Source: Environ Health. 2016 Apr 12;15:50. doi: 10.1186/s12940-016-0131-2 (PMC4828888; doi:10.1186/s12940-016-0131-2)
Supplement: Additional file 3: — Long-term dietary exposure to potential sources of BPA, and correlation with urinary BPA in Conjugated Form1 and uBPA in Second Trimester Pregnant Women (DOCX 21 kb) [file 12940_2016_131_MOESM3_ESM.docx]

**Direct measurement of Bisphenol A (BPA), BPA glucuronide and BPA sulfate in a diverse and low-income population of pregnant women reveals high exposure, with potential implications for previous exposure estimates: a cross-sectional study.**

*Roy R. Gerona, Janet Pan, Ami R. Zota, Jackie M. Schwartz, Matthew Friesen, Julia A. Taylor, Patricia A. Hunt, Tracey J. Woodruff*

# Additional File 3

# Table 1. Long-term dietary exposure to potential sources of BPA, and correlation with urinary BPA in Conjugated Form^1^ and uBPA in Second Trimester Pregnant Women, Northern and Central California, 2009-2011 (n=112)

|  |  |  | **Spearman ρ** | |
| --- | --- | --- | --- | --- |
|  | **Median** | **Range** | **BPA in Conjugated Form^1^** | **uBPA** |
| Canned Drinks (cans/week) | 4.75 | 0 – 126 | -0.05 | 0.09 |
| Canned Foods (cans/week) | 4.75 | 0 – 110.75 | -0.15 | 0.03 |
| Carton Drinks (cartons/week) | 0.5 | 0 – 84 | -0.11 | -0.06 |
| Drinks served in Paper Cups (cups/week) | 1 | 0 – 56.5 | -0.12 | -0.03 |
| Foods wrapped in or served on paper or cardboard (items/week) | 4.5 | 0 – 71 | -0.16^*^ | 0.04 |

* p <0.05

1. Conjugated BPA=BPA in glucuronide form + BPA in sulfate form. BPA in glucuronide form=BPA glucuronide*0.5614. BPA in sulfate form=BPA sulfate*0.7404. The factors 0.5614 and 0.7404 are the ratios of the molecular weight of BPA to the molecular weights of BPA glucuronide and BPA sulfate, respectively.
