# Supplementary material for: Direct measurement of Bisphenol A (BPA), BPA glucuronide and BPA sulfate in a diverse and low-income population of pregnant women reveals high exposure, with potential implications for previous exposure estimates: a cross-sectional study
Source: Environ Health. 2016 Apr 12;15:50. doi: 10.1186/s12940-016-0131-2 (PMC4828888; doi:10.1186/s12940-016-0131-2)
Supplement: Additional file 4: — University of Missouri Laboratory Confirmation of BPA glucuronide: Laboratory Methods (DOCX 25 kb) [file 12940_2016_131_MOESM4_ESM.docx]

**Direct measurement of Bisphenol A (BPA), BPA glucuronide and BPA sulfate in a diverse and low-income population of pregnant women reveals high exposure, with potential implications for previous exposure estimates: a cross-sectional study.**

*Roy R. Gerona, Janet Pan, Ami R. Zota, Jackie M. Schwartz, Matthew Friesen, Julia A. Taylor, Patricia A. Hunt, Tracey J. Woodruff*

# Additional File 4

# University of Missouri Laboratory Confirmation of BPA glucuronide: Laboratory Methods

**Materials**

*Reagents.* Solvents and water were HPLC grade, and were obtained from Fisher Scientific. BPA was obtained from Sigma-Aldrich (St. Louis MO; purity >99%). C^13^-BPA was obtained from Cambridge Isotope Laboratories Inc. (Andover, MA; purity 99%), and both BPA glucuronide (purity 98%) and C^13^-BPA -glucuronide (BPA-DG; purity >99%) were provided by the National Institute of Environmental Health Sciences (NIEHS), Research Triangle Park, NC. C^13^-BPA was obtained from Cambridge Isotope Laboratories (Tewksbury, MA), and d6-BPA was obtained from C/D/N Isotopes Inc. (Pointe-Claire, Quebec, Canada.)

**Extraction of BPA and BPA conjugates in urine.**

a) For direct measurements of uBPA and BPA glucuronide, the six urine samples were passed through a Thermo Hypersep C18 SPE cartridge as described elsewhere [1]. Samples were spiked with C^13^- BPA glucuronide and d6-BPA as internal standards. Recoveries of these internal standards averaged 93% and 107% respectively.

b) For measurement of Total BPA after enzyme hydrolysis, samples were first spiked with the C^13^-BPA glucuronide and then incubated in 100 mM ammonium acetate buffer (pH 5.0) overnight, with either 1000 units b-glucuronidase Type H-1 or 2U of units b-glucuronidase Type H-3 (both enzymes from Sigma); preliminary tests indicated equal effectiveness of the two preparations at these concentrations. The hydrolyzed samples were spiked with d6-BPA as an internal standard, and then extracted twice with methyl tert-butyl ether as described elsewhere [2]. The extract was dried under nitrogen and reconstituted in 50:50 methanol:H2O for HPLC. The total BPA concentration (representing a combined measure of deconjugated BPA conjugates and free BPA) was measured by LC/MSMS. The measured average recovery of d6-BPA was 86%. Recovery of C^13^-BPA (resulting from deconjugation of the C^13^- BPA glucuronide internal standard) averaged 92% of expected values across three independent experiments.

**LC/MSMS methods***.*

uBPA and BPA glucuronide in urine (either with or without prior hydrolysis) were quantified by liquid chromatography with mass spectrometry (LC-MS/MS) using a Thermo TSQ Quantum Access Max (Thermo Fisher Scientific, Waltham, MA) connected to an integrated Thermo-Accela LC system, as described elsewhere [1]. Analytes were detected using electrospray ionization with negative polarity, and conditions (tube lens setting, collision energy) were optimized for each analyte using the instrument software. Separations were performed on a 50x2.1 mm 2.6 micron Kinetix HPLC column (Phenomenex, Torrance, CA), at a flow rate of 200 µL/min. A gradient mobile phase was employed using 20:80 methanol:water and methanol. Thermo LCQuant software was used to autotune, acquire, and process the LC/MS data. BPA, C^13^-BPA, BPA glucuronide and C^13^- BPA glucuronide were detected using selected reaction monitoring for m/z 227>212, m/z 239>224, m/z 403>227 and 415>239 respectively, and quantitation was made against standard curves of the analytes at concentrations ranging from 1-100 ng/ml. The limit of detection and limit of quantitation (LOD and LOQ) were defined respectively as 3X and 10X the standard deviation of the lowest concentration in the standard curve. The LOD for uBPA and BPA glucuronide were 0.41 and 0.36 ng/mL respectively, and the LOQ for uBPA and BPA glucuronide were 1.19 and 1.36 ng/mL, respectively.

**References**

1. Vandenberg, L.N., et al., *A round robin approach to the analysis of bisphenol a (BPA) in human blood samples.* Environ Health, 2014. **13**(1): p. 25.

2. Taylor JA, v.S.F., Welshons WV, Drury B, Rottinghaus G, Hunt PA, et al., *Similarity of bisphenol A pharmacokinetics in rhesus monkeys and mice: relevance for human exposure.* Environ Health Perspect, 2011. **119**: p. 422-430.
